# *P. brasiliensis* Virulence Is Affected by SconC, the Negative Regulator of Inorganic Sulfur Assimilation

**DOI:** 10.1371/journal.pone.0074725

**Published:** 2013-09-16

**Authors:** João Filipe Menino, Margarida Saraiva, Jéssica Gomes-Rezende, Mark Sturme, Jorge Pedrosa, António Gil Castro, Paula Ludovico, Gustavo H. Goldman, Fernando Rodrigues

**Affiliations:** 1 Life and Health Sciences Research Institute (ICVS), School of Health Sciences, University of Minho, Braga, Portugal; 2 ICVS/3 B’s - PT Government Associate Laboratory, University of Minho, Braga/Guimarães, Portugal; 3 Laboratório Nacional de Ciência e Tecnologia do Bioetanol, CTBE, Campinas, São Paulo, Brasil; 4 Faculdade de Ciências Farmacêuticas de Ribeirão Preto, Universidade de São Paulo, São Paulo, Brasil; Instituto de Salud Carlos III, Spain

## Abstract

Conidia/mycelium-to-yeast transition of *Paracoccidioides* *brasiliensis* is a critical step for the establishment of paracoccidioidomycosis, a systemic mycosis endemic in Latin America. Thus, knowledge of the factors that mediate this transition is of major importance for the design of intervention strategies. So far, the only known pre-requisites for the accomplishment of the morphological transition are the temperature shift to 37°C and the availability of organic sulfur compounds. In this study, we investigated the auxotrophic nature to organic sulfur of the yeast phase of *Paracoccidioides* , with special attention to *P. brasiliensis* species. For this, we addressed the role of SconCp, the negative regulator of the inorganic sulfur assimilation pathway, in the dimorphism and virulence of this pathogen. We show that down-regulation of *SCONC* allows initial steps of mycelium-to-yeast transition in the absence of organic sulfur compounds, contrarily to the wild-type fungus that cannot undergo mycelium-to-yeast transition under such conditions. However, *SCONC* down-regulated transformants were unable to sustain yeast growth using inorganic sulfur compounds only. Moreover, pulses with inorganic sulfur in *SCONC* down-regulated transformants triggered an increase of the inorganic sulfur metabolism, which culminated in a drastic reduction of the ATP and NADPH cellular levels and in higher oxidative stress. Importantly, the down-regulation of *SCONC* resulted in a decreased virulence of *P. brasiliensis*, as validated in an *in vivo* model of infection. Overall, our findings shed light on the inability of *P. brasiliensis* yeast to rely on inorganic sulfur compounds, correlating its metabolism with cellular energy and redox imbalances. Furthermore, the data herein presented reveal SconCp as a novel virulence determinant of *P. brasiliensis*.

## Introduction

*Paracoccidioides* *brasiliensis* is a dimorphic fungus and a causative agent of paracoccidioidomycosis, an endemic mycosis affecting the population from Latin America countries such as Brazil, Colombia and Venezuela [1]. The infective process comprises a temperature-dependent morphological switch of the fungus from the conidia/mycelium phase at environmental temperatures (around 26°C) to the pathogenic yeast phase at the mammalian host temperature (around 37°C) [1,2]. In addition to the well-studied temperature requisite, the knowledge of other regulators mediating both morphogenesis and virulence in *P. brasiliensis* is scarce. Thus, a better understanding of *P. brasiliensis* metabolic processes is essential to unravel its virulence determinants, offering novel targets for prophylaxis and/or therapeutics intervention.

Several studies show that sulfur metabolism is directly correlated with virulence of bacteria and dimorphic fungi like *Histoplasma capsulatum* [3-6]. Sulfur compounds play a role in the formation of functional thiol (-SH) groups, crucial for innumerous cellular components and signaling processes [7,8]. Despite the relevance of sulfur as constituent of essential organic molecules such as coenzyme-A and glutathione [9], the preferential pathway of sulfur assimilation, from either organic or inorganic compounds, in pathogenic fungi is not completely understood. Sulfur requirements of *P. brasiliensis* differ between the mycelium and the yeast phase of the fungus. Similarly to other dimorphic fungi, the growth of *P. brasiliensis* in the yeast phase depends on organic sulfur sources [10-12]. In contrast, and despite being auxotrophic for organic sulfur compounds such as cysteine or methionine, *P. brasiliensis* is prototrophic in the mycelial phase [10-12].

Recent transcriptomic approaches revealed that genes related to sulfur metabolism are differentially expressed in the mycelium and yeast phase of *P. brasiliensis* [10,13]. In particular, expression of inorganic sulfur metabolism-related genes was markedly up-regulated in the yeast phase of this fungus [13-17]. For example, a 35-fold increase in the expression of *METR*, the positive regulator of the inorganic sulfur assimilation pathway, was observed [13]. Another differentially expressed gene is *SCONC*, which as *METR* is highly expressed in the yeast phase and down-regulated in the mycelial phase of *P. brasiliensis* [13]. *SCONC* gene encodes a negative regulator of the inorganic sulfur assimilatory pathway, orchestrating the repression of genes from the sulfur assimilatory pathway by promoting MetR proteolysis [18,19]. However, the low levels of *SCONC* in the mycelial phase allow *P. brasiliensis* to use inorganic sulfur. Therefore, the cross-talk of these two regulators has been suggested to be responsible for the yeast-phase auxotrophy for organic sulfur compounds, by preventing the assimilation of inorganic sulfur by the pathogenic yeast cells. Although the molecular bases underlying the distinct sulfur requirements of *P. brasiliensis* are presently more elucidated, the physiological aspects behind the sulfur-dependent dimorphic behavior are far from being understood. A recent transcriptome study showed that *SCONC* was being highly expressed in *P. brasiliensis* yeast cells recovered from infected mice than in those cultured *in vitro* [20]. Therefore, a stronger repression of inorganic sulfur assimilation seems to be in place during infection, indicating that yeast cells alter their metabolism *in vivo*, most probably due to higher access to organic sulfur. It is likely that this change plays a role in the fungus pathogenesis. For these reasons, a better understanding of the impact played by SconCp on the sulfur-dependent dimorphic processes in *P. brasiliensis* is crucial, as it may highlight a new *P. brasiliensis* virulence factor.

To address these questions, we down-regulated the expression of *SCONC* in isolates from different *Paracoccidioides* species and investigated its impact both on the inorganic sulfur assimilatory pathway and on the dimorphic transition. In addition, we evaluated the role of SconCp as a possible *P. brasiliensis* virulence factor, using an *in vivo* mouse model of infection. We herein present evidence that *P. brasiliensis* SconCp acts as a regulator of dimorphism by modulating the inorganic sulfur metabolism, thereby influencing the virulence of this pathogenic fungus. 

## Materials and Methods

### Microorganisms and culture media

*Paracoccidioides* wild-type species and *SCONC* down-regulated strains are listed in Table 1. Yeast cells were maintained at 37°C by subculturing in brain heart infusion (BHI) (Duchefa) solid media supplemented with 1% glucose and gentamicin (50 µg/mL). For the expression studies, *Paracoccidioides* yeast cells were grown in synthetic McVeigh Morton (MMvM) liquid medium [21] at 37°C with aeration on a mechanical shaker (220 rpm). For the *in vivo* assays, yeast cells were grown in BHI liquid medium supplemented with 1% glucose and gentamicin (50 µg/mL) at 37°C with aeration on a mechanical shaker (220 rpm). Cell growth was monitored for 148 h by microscopic counting using a Neubauer counting chamber and cells were collected during the exponential growth phase (72 h of growth, 1.65 ± 0.8 x 10^7^ cells/mL) for the infection.

**Table 1:** *Paracoccidioides* species used in this study.

**Phylogenetic group**	**Isolate**	**Location**	**Source**		**Reference**
*P. lutzii*	Pb01	Brazil	Chronic PCM		[47]
*P. lutzii*	Pb01 As*SCONC* D	N.A.*	This study
PS3	Pb60855	Colombia	Chronic PCM		[48]
PS3	Pb60855 As*SCONC* A	N.A.*	This study		
PS3	Pb60855 As*SCONC* B	N.A.*	This study		

* N.A. – Not applicable

*Agrobacterium tumefaciens* strain LBA1100 [22] was used as the recipient for the binary vector constructed in this study. Bacterial cells were maintained at 28°C in Luria Bertani (LB) medium containing kanamycin (100 µg/ml). *Escherichia coli* JM109 competent cells (Promega) were grown at 37°C in LB medium supplemented with appropriate antibiotics and were used as the host for plasmid amplification and cloning.

For the morphological transition, complete MMvM (MMvM +Cys/+SO_4_^2-^) [supplemented with L-cysteine (1.7 mM) as organic sulfur source and MgSO_4_.7H_2_O (2 mM) and (NH_4_)_2_SO_4_ (15 mM) as inorganic sulfur sources], MMvM without inorganic sulfur compounds supplementation (MMvM +Cys/-SO_4_^2-^) and MMvM without organic sulfur compounds supplementation (MMvM -Cys/+SO_4_^2-^) were used. Briefly, for the yeast-to-mycelium transition yeast cells were cultured at 37°C to the exponential growth phase in complete MMvM, washed 3 times with sterile phosphate-buffered saline (PBS), and inoculated at a final concentration of 1x10^6^ cells/mL in the appropriated medium. The cultures were then transferred to a mechanical shaker (220 rpm) at 26°C and cultured until complete transition was accomplished. For the mycelium-to-yeast transition, mycelium was cultured at 26°C in complete MMvM medium, and washed 3 times with sterile PBS. The cultures were then transferred to a mechanical shaker (220 rpm) at 37°C and cultured till no more morphological changes were being observed.

### Construction of *Paracoccidioides* *SCONC* Antisense-RNA (As*SCONC*) Isolates

**Figure 1:**
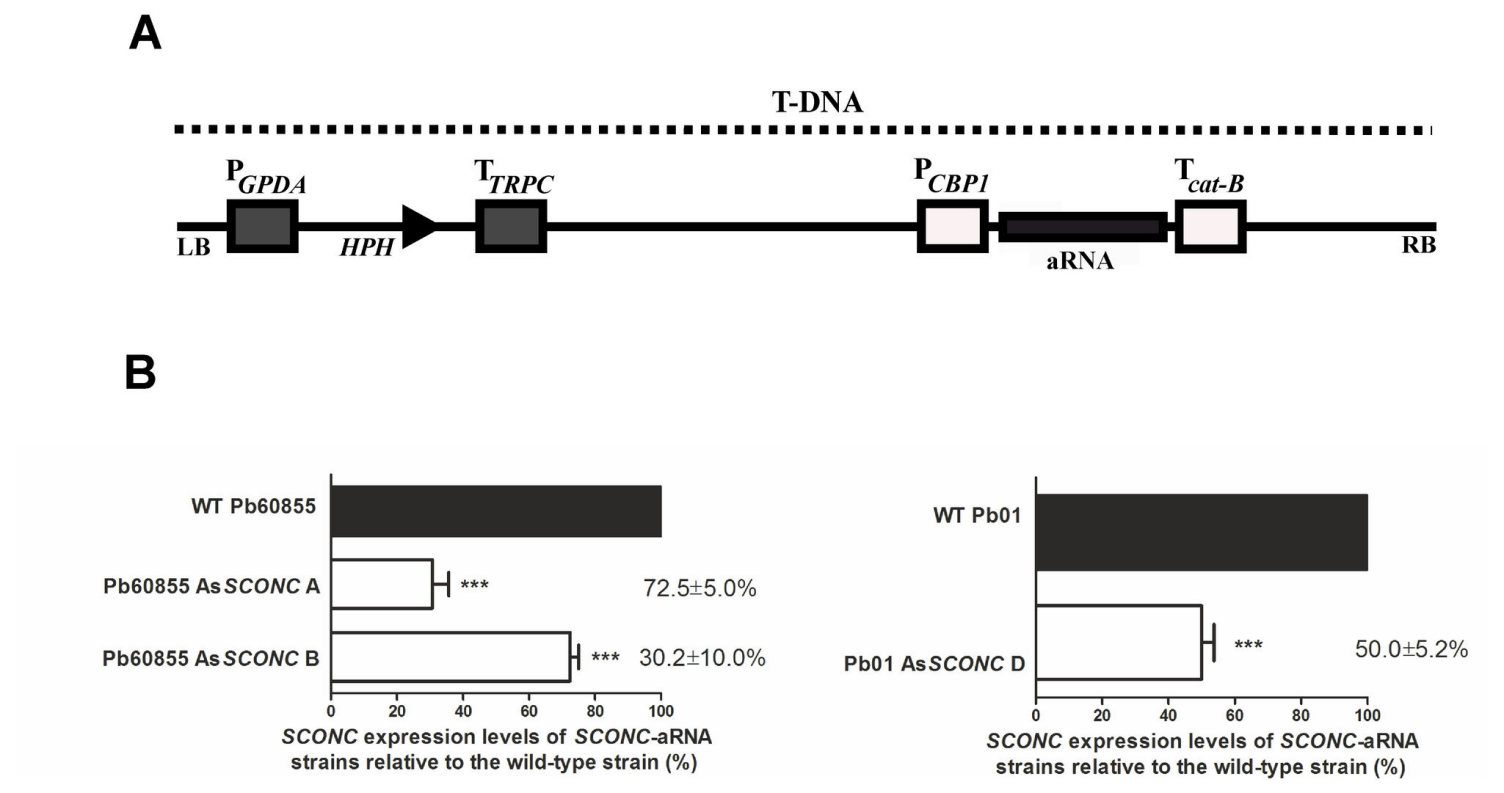
Silencing of *SCONC* expression by targeted antisense RNA in *Paracoccidioides* species decreases mRNA levels. (**A**) Structure of aRNA Transfer DNA (T-DNA) cassette inserted into *P. brasiliensis* yeast cells via ATMT in order to silence *SCONC*. The antisense RNA (As*SCONC*) sequence was placed under control of the calcium binding protein promoter from *H* *. capsulatum* (CBP1) with hygromycin B phosphotransferase (HPH) gene under control of the glyceraldehyde 3-phosphate promoter from *A. nidulans* (PGPDA). (**B**) Gene expression levels of *SCONC* in *Paracoccidioides* clones harboring silencing oligonucleotides targeting the *SCONC* coding sequence compared to the respective wild-type strain. Cells were grown to the exponential phase in complete MMvM (MMvM +Cys/+SO_4_^2-^) at 37°C and collected for gene expression evaluation. Asterisks represent statistical differences between wild-type and down-regulated strains (***p<0.001). Percentage of gene expression levels reduction was obtained comparing *SCONC* levels of each clone to those of the wild-type strains. *SCONC* expression levels determined by qRT-PCR were normalized to expression of the internal reference gene β-tubulin (TUB2). Bars represent means and standard deviations.

Plasmid DNA extraction, recombinant DNA manipulations, and *E. coli* transformation procedures were performed as described elsewhere [23]. *P. brasiliensis* wild-type strain ATCC 60855 DNA was extracted from yeast cultures during exponential growth and a high-fidelity proof-reading DNA polymerase (NZYTech) was employed to amplify an aRNA oligonucleotide sequence targeting the coding sequence of *SCONC*. The As*SCONC* sequence was inserted into the plasmid pCR35 under the control of the calcium-binding protein (CBP1) promoter region from *H* *. capsulatum* as previously described [24]. The aRNA cassette was subsequently cloned into the transfer DNA (T-DNA) region of the binary vector pUR5750 (Figure 1.A) and mobilized to *A. tumefaciens* LBA1100 ultracompetent cells by electroporation as previously described [23]. Transformants were isolated by selection on kanamycin at 100 µg/ml.

Insertion of recombinant T-DNA harboring the As*SCONC* cassette and a hygromycin B resistance marker into the genome of *Paracoccidioides* yeast cells was accomplished by *A. tumefaciens*-mediated transformation (ATMT) [23]. Briefly, *A. tumefaciens* LBA1100 carrying binary vector pUR5750 harboring the As*SCONC* sequence was grown overnight in LB liquid medium with antibiotics at 28°C with agitation. Bacterial cells were spun down, washed, set to an OD_660nm_ of 0.30 in induction medium (IM) [25] with acetosyringone (Sigma, USA) (200µM), and re-incubated at 28°C until an OD_660nm_ of approximately 0.80. *Paracoccidioides* yeast cells were grown in BHI batch cultures to the exponential growth phase and cells were washed with IM and adjusted to a final concentration of 1x10^8^ cells/ml using direct microscopic counts (Neubauer counting chamber procedures). A 1:10 *A. tumefaciens*/*Paracoccidioides* ratio was inoculated onto sterile Hybond N membrane (Amersham Biosciences, USA) on solid IM for co-cultivation at 25°C for 3 days. Prior to incubation, co-cultivation plates with cellular mixtures were air dried in a safety cabinet for 30 min. Following co-cultivation, membranes were transferred to BHI liquid medium containing cefotaxime (200 µg/ml), cells dislodged by aid of a spatula, and the cell suspension incubated for 48 h at 36°C, 200 rpm, before plating on selective BHI media (HygB 75 µg/ml). Selection plates were monitored for colony forming ability at 36°C for 15 days. Randomly selected HygB resistant transformants confirmed by PCR were tested for mitotic stability and selected for further assays. An identical procedure was followed to obtain the *Paracoccidioides* *lutzii* Pb01 As*SCONC* transformant.

### Quantitative real-time polymerase chain reaction (qRT-PCR)

*Paracoccidioides* yeast cultures were inoculated from a single colony and grown to exponential phase in complete MMvM at 37°C (200 rpm). Medium was refreshed once after 4 days. Total RNA (1 µg) from wild-type and As*SCONC* transformants was isolated according to TRIzol protocol (Invitrogen, USA) and RNA samples were subsequently treated with 2U of DNaseI (Ambion, USA) by incubation for 1 h at 37°C. The absence of DNA contamination in the samples was confirmed by the lack of PCR amplification of the *GP43* gene in the isolated RNA. Total RNA (1 µg) was reverse transcribed using the iScript cDNA Synthesis kit (Bio-Rad, France) following manufacturer’s instructions and 1 µL of cDNA used as a template for real-time quantification using the SsoFast EvaGreen SuperMix (Bio-Rad, France) following manufacturer’s instructions. Real-time quantification was carried out on a CFX96 Real-Time System (Bio-Rad, France) using threshold cycle (Ct) values for β-tubulin (*TUB2*) transcripts as the endogenous reference. The primer sequences were designed and synthesized by NZYTech and are described in Table 2. All measurements were performed in triplicate. A single melting peak was obtained for each gene analyzed in all samples.

**Table 2:** List of primers used in this study.

**Gene name**	**Sequence (5' to 3'**)
β-tubulin (*TUB2*)	**Forward** *aga aca tga tgg ctg ctt cc*
	**Reverse** *gcg cat ctg atc ttc gac ttc*
Sulfur controler (*SCONC*)	**Forward** *gaa tgg tgc gaa cat cac ag*
	**Reverse** *cca gga tta tct caa aaa gc*
bZIP transcription factor (*METR*)	**Forward** *ttc ttg agc cac cga ttc tcc*
	**Reverse** *gga gcg cac cgt taa gga g*
Sulfate permease (SP)	**Forward** *tgg tca gtt ggc ttg tga ac*
	**Reverse** *tta gca tca acc tgg gga ac*
Choline-O sulfatase (*CHS*)	**Forward** *tga aca acg ctt gac cag tg*
	**Reverse** *cgg aag aca tat cat ggt acc*
ATP sulfurylase (*MET3*)	**Forward** *cgt tgg agg aaa ggt tga ag*
	**Reverse** *ctc gat gca tgg gat tta tc*
PAPS reductase (*MET16*)	**Forward** *cca att cct aga acc gca ag*
	**Reverse** *gag ttt gga gag cat gtc gag*
Sulfite reductase (*MET10*)	**Forward** *ccc acc gat atc cat acc ac*
	**Reverse** *tcc ata ggc ctc ctt gaa ga*

### Microscopy

To evaluate cell morphology during the dimorphic processes, four morphotypes were determined according to Nunes and co-workers (2005) [19]. *Paracoccidioides* cells were collected during the transition process (yeast-to-mycelium or mycelium-to-yeast) in complete MMvM, MMvM +Cys/-SO_4_^2-^ and MMvM -Cys/+SO_4_^2-^ and fixed as previously described [26]. A total of 300 morphological units were counted for each culture (in triplicate), and the estimation of each morphological state (from yeast to mycelium and vice-versa) was performed in terms of percentage. Morphological units were counted using a Zeiss Axioskop equipped with a Carl Zeiss AxioCam (Carl Zeiss, Jena).

### Determination of *P. brasiliensis* growth curve in yeast cells

Yeast cells of *P. brasiliensis* wild-type Pb60855 and As*SCONC* transformants were grown in MMvM, MMvM +Cys/-SO_4_^2-^ and MMvM -Cys/+SO_4_^2-^ at 37°C and samples were collected to determine cell number, using a standard Neubauer system, at different times until stationary growing phase was reached. All cultures were started from an initial concentration of 1x10^5^ cells/mL.

### ATP/NADPH measurements

ATP measurements were performed according to [27]. Briefly, cells were collected by centrifugation and the pellet was frozen with liquid nitrogen and stored at -80°C. For the measurements, the pellet was mixed with 200 µL of 5% trichloroacetic acid (TCA) and vortexed twice for one minute, with one minute interval on ice. The mix was centrifuged for 1 min at 4°C and 10 µL of the supernatant were added to 990 µL of reaction buffer (25 mM HEPES, 2 mM EDTA, pH 7.75). Of this mixture, 100 µL were added to 100 µL of Enliten Luciferin/Luciferase Reagent (Promega) and luminescence was measured on a ThermoScientific Fluoroskan Ascent FL.

NADPH measurements were performed according to manufacturer’s instructions (NADP/NADPH Assay Kit, Abcam). Briefly, cells were collected by centrifugation and washed with ice-cold PBS. After pelleting 2x10^5^ cells for each sample, 400 µL of NADP/NADPH extraction buffer was added. Two freeze/thaw cycles (20 min on dry-ice followed by 10 min at room-temperature) were applied, and after vortexing for 10 sec the supernatant was collected. Of each supernatant, 50 µL were incubated with 100 µL of NADP cycling mix for 5 min at room temperature. Next step, 10 µL of NADPH developer were added to each sample and the mixture was incubated at room temperature for 1 hour. Total NADP (NADP^+^ + NADPH) and NADPH only were measured at OD_450nm_ on a Bio-Rad 680 Micro-plate Reader.

### Pulse with inorganic sulfur sources

*P. brasiliensis* wild-type Pb60855 and As*SCONC* yeast strains were cultured to the exponential phase of growth in complete MMvM (MMvM +Cys/+SO_4_^2-^) at 37°C (200 rpm). Cells were collected, washed three times in sterile PBS, and transferred to MMvM without inorganic sulfur compounds supplementation (MMvM +Cys/-SO_4_^2-^) to a final concentration of 1x10^6^ cells/mL. A pulse with inorganic sulfur sources [MgSO_4_.7H_2_O (final concentration 2 mM) and (NH_4_)_2_SO_4_ (final concentration 15 mM)] was given to all the cultures, and samples were collected 15 and 30 min after the pulse for posterior analysis.

### Measurement of reactive oxygen species

Intracellular reactive oxygen species (ROS) were detected by dihydrorhodamine (DHR-123) or dihydroethidium (DHE) (Molecular Probes) staining. For evaluation of H_2_O_2_ levels, cells were incubated with 15 mg/mL of DHR-123 for 90 min at 30°C in the dark, washed with PBS and measured by flow cytometry. For evaluation of O_2_^-^ levels, cells were incubated with 5 mM DHE for 10 min at 30°C in the dark, washed with PBS and evaluated by flow cytometry. All measurements were performed in a BD™ LSR II flow cytometer. A minimum of 100,000 cells per sample was acquired at low/medium flow rate. Offline data was analyzed with the flow cytometry analysis software package FlowJo 7.6.1.

### In vivo infection

Eight-week-old C57BL/6 male mice were obtained from Charles River (Barcelona, Spain). Mice were housed under specific-pathogen-free conditions with food and water *ad libitum*. C57BL/6 WT mice were infected intravenously (i.v.) with 1x10^6^ *P. brasiliensis* yeast cells grown to the exponential phase in BHI liquid medium (either wild-type Pb60855 or each of the corresponding As*SCONC* transformants). Prior to infection, cells were washed 3 times with lipopolysaccharide (LPS)-free PBS (Gibco), passed through a syringe to eliminate cell clumps, and submitted to Neubauer counting procedures (each mother and bud cells were considered as individual counts). Mice survival was monitored for 80 days.

### Ethics statement

This study was approved by the Portuguese national authority for animal experimentation Direção Geral de Veterinária (ID: DGV 594 from 1st June 2010). Animals were kept and handled in accordance with the guidelines for the care and handling of laboratory animals in the Directive 2010/63/EU of the European Parliament and of the Council.

### Statistics

Data are reported as the mean ± standard error of the mean (SEM) and all assays were repeated at least three times. All statistical analyses were performed using the GraphPad Prism Software version 5.01. For the experiments comparing two groups, a two-tailed unpaired Student *t* test was performed. Welch’s correction was applied when making multiple comparisons. The survival curves, representative of two independent experimental infections (n=20 mice), were compared using the Chi square Logrank Test. For all data analysis statistical significance was considered at the level of 0.05 (2-tailed, 95% confidence interval).

## Results

### Silencing of the *Paracoccidioides* *SCONC* gene leads to the up-regulation of genes involved in the inorganic sulfur metabolism

**Figure 2:**
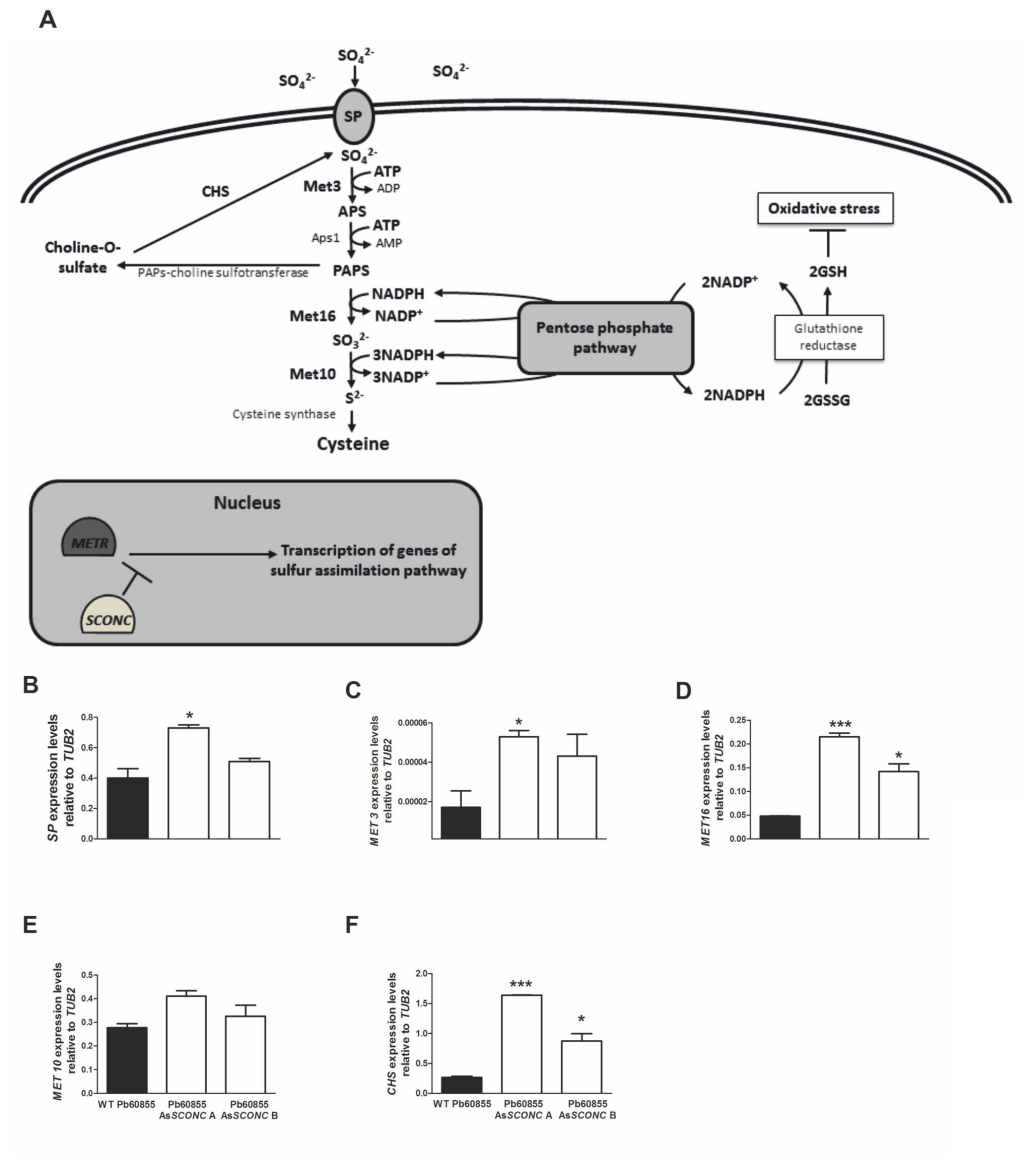
Sulfur assimilatory pathway and interplay with the glutathione system and pentose phosphate pathway. (**A**) SconCp orchestrates MetRp proteolysis, therefore resulting in the transcriptional repression of several genes from this pathway. The interplay between sulfur assimilatory pathway and the pentose phosphate pathway for the inter-conversion of NADP^+^ and NADPH will impact on the levels of reduced glutathione (GSH), therefore on cells redox balance. (**B**-**F**) *SCONC* down-regulation results in increased expression levels of inorganic sulfur assimilation pathway related genes. Expression profiles of genes coding sulfate permease (SP), ATP sulfurylase (MET 3), PAPS reductase (MET 16), sulfite reductase (MET 10) and choline-O sulfatase (CHS) in wild-type strain Pb60855 and two down-regulated clones, Pb60855 As*SCONC* A and Pb60855 As*SCONC* B grown to the exponential phase in complete MMvM (MMvM +Cys/+SO_4_^2-^) at 37°C. Asterisks represent statistical differences between wild-type strain and aRNA clones (*p<0.05; ***p<0.001). *SCONC* expression levels determined by qRT-PCR were normalized to expression of the internal reference gene β-tubulin (TUB2). Bars represent means and standard deviations.

To evaluate the impact of *SCONC* down-regulation in the yeast phase of *P. brasiliensis*, we constructed an antisense RNA sequence targeting the coding sequence of the *SCONC* gene (Figure 1.A) for genome integration using ATMT methodology [23,26]. Two independent *SCONC* down-regulated transformants for *P. brasiliensis* wild-type 60855 (Pb60855 As*SCONC* A and Pb60855 As*SCONC* B) were selected. Due to the yet impossible gene disruption and complementation in *P. brasiliensis* [23,26], we replicated our findings by down-regulating *SCONC* in the strain Pb01 of *Paracoccidoideslutzii* . For this strain one clone (Pb01 As*SCONC* D) was also selected for analysis. The percentage of reduction in the expression levels of *SCONC* in the As*SCONC* transformants was calculated by comparison to expression levels in the respective wild-type strains (Figure 1.B). For Pb60855 As*SCONC* A and Pb60855 As*SCONC* B the percentages of *SCONC* down-regulation were around 73% and 30%, respectively, whereas for Pb01 As*SCONC* D it was around 50%. To evaluate the impact of *SCONC* silencing in the inorganic sulfur assimilatory pathway (Figure 2.A), we analyzed the expression levels of several genes described to be involved in this pathway [18] using exponential yeast cells cultured in complete MMvM. Although similar differences in the expression of the targeted genes were found for both transformants As*SCONC* A and B, differences for As*SCONC* A were consistently more pronounced, probably due to the better efficiency of *SCONC* silencing in this transformant. The expression of the gene encoding sulfate permease (SP), the membrane transporter responsible for inorganic sulfate uptake in *P. brasiliensis* [13], was found to be significantly up-regulated in Pb60855 As*SCONC* A when compared to the wild-type strain (Figure 2.B). Also up-regulated in the As*SCONC* transformants was the expression of several downstream genes of the inorganic sulfur assimilatory pathway (Figure 2.C-E) and that of choline sulfatase (*CHS*) (Figure 2.F), an enzyme of the lateral branch of the inorganic sulfur assimilation pathway, that uses as substrate choline-O-sulfate, a osmoprotectant and an additional intracellular source of inorganic sulfur [18]. Overall, our data is in line with previous reports [13,28-30] showing a key role for *SCONC* as a negative transcriptional regulator of the inorganic sulfur metabolism.

### *SCONC* down-regulation promotes mycelium-to-yeast transition in the absence of organic sulfur sources, but does not support yeast growth

**Figure 3:**
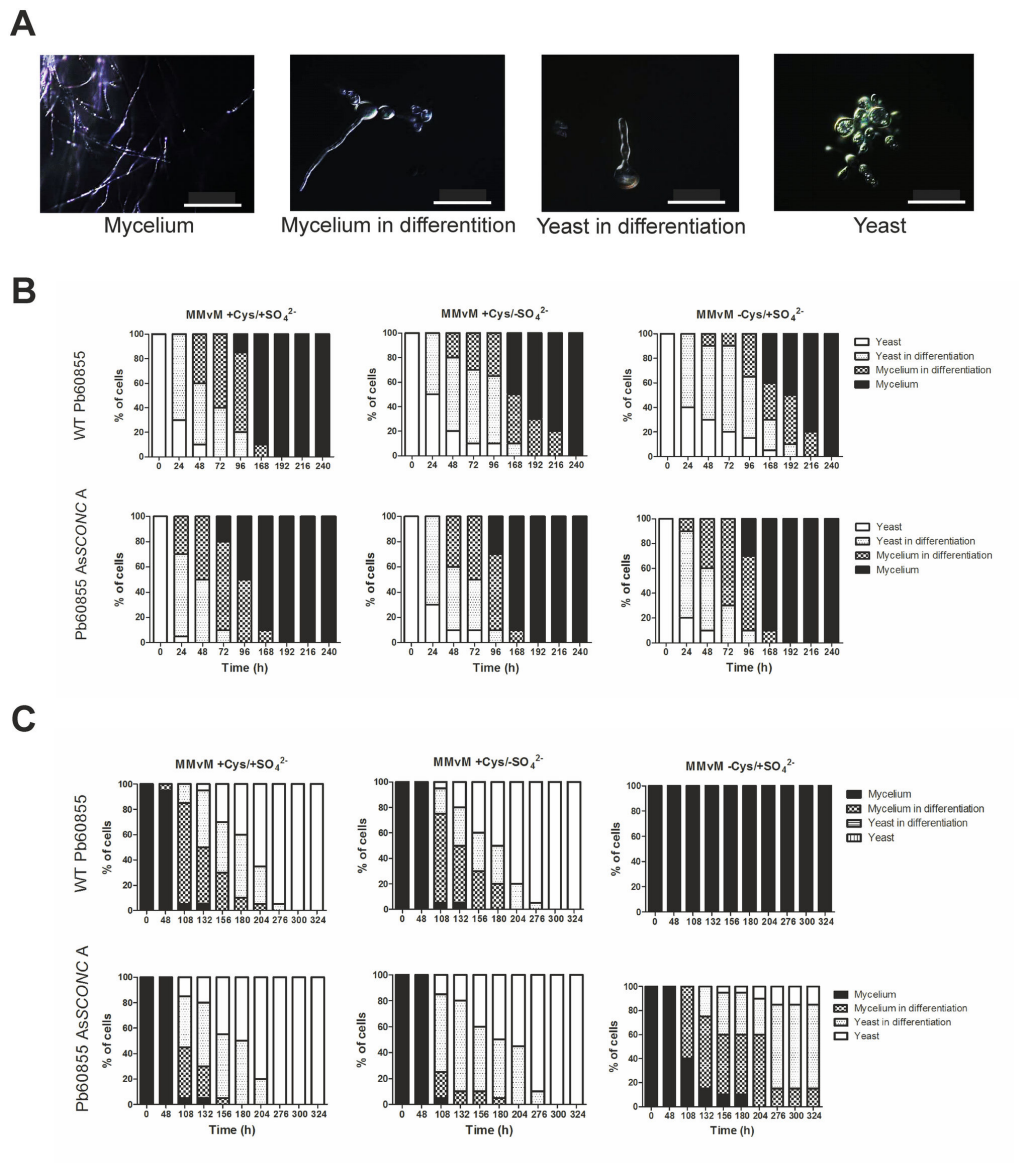
Down-regulation of *SCONC* in *P. brasiliensis* allows mycelium-to-yeast transition in the absence of organic sulfur compounds. (**A**) Representative DIC pictures of each morphotype considered during the morphological transition. Magnification: x40 (White bars represent 200 µm). (**B**) Evaluation of Pb60855 and Pb60855 As*SCONC* A morphotypes during yeast-to-mycelium transition at 26°C in complete MMvM (MMvM +Cys/+SO_4_^2-^), MMvM without inorganic sulfur compounds supplementation (MMvM +Cys/-SO_4_^2-^) and MMvM without organic sulfur compounds supplementation (MMvM -Cys/+SO_4_^2-^); (**C**) Evaluation of Pb60855 and Pb60855 As*SCONC* A morphotypes during mycelium-to-yeast transition at 37°C in complete MMvM (MMvM +Cys/+SO_4_^2-^), MMvM without inorganic sulfur compounds supplementation (MMvM +Cys/-SO_4_^2-^) and MMvM without organic sulfur compounds supplementation (MMvM -Cys/+SO_4_^2-^).

Considering that *Paracoccidoides* conidia/mycelium-to-yeast morphological switch is a critical step in the infective process [1] and that the high expression of *SCONC* may be responsible for the organic-sulfur auxotrophy of the yeast phase [13], we next investigated the effects of *SCONC* down-regulation on both transitions yeast-to-mycelium and mycelium-to-yeast. To address the yeast-to-mycelium transition, we cultured yeast cells of *Paracoccidioides* wild-type and As*SCONC* transformants at 26°C in three different media: complete MMvM, MMvM without inorganic sulfur compounds supplementation (MMvM +Cys/-SO_4_^2-^) and MMvM without organic sulfur compounds supplementation (MMvM -Cys/+SO_4_^2-^). The presence of each morphotype (Figure 3.A) during the transition process was evaluated over time. As expected, the yeast-to-mycelium transition was successfully accomplished in both wild-type strains (Pb60855 and Pb01) and in the respective As*SCONC* transformants, in complete medium or in the absence of either inorganic or organic sulfur compounds (Figure 3.B and Figures S1.A and S2.A). To investigate the mycelium-to-yeast transition, we cultured mycelium of wild-type strains and As*SCONC* transformants in the above mentioned media at 37°C. Both Pb60855 and Pb01 wild-type strains were able to completely convert to the yeast phase in complete MMvM and MMvM without inorganic sulfur supplementation (Figure 3.C and Figure S2.B). The same was observed for all the As*SCONC* transformants (Figure 3.C and Figures S1.B and S2.B). However, wild-type strains were unable to switch from the mycelium to the yeast phase or other intermediate phases in MMvM without organic sulfur (Figure 3.C and Figure S2.B). These results confirmed the auxotrophy of the yeast phase of *Paracoccidioides* species for organic sulfur compounds, as previously reported [10,13]. Conversely, the morphological switch of the As*SCONC* transformants to the yeast phase in MMvM without organic sulfur was compromised, occurring at a lower efficiency (Figure 3.C and Figures S1.B and S2.B). For all As*SCONC* transformants, the cultures under transition contained a considerable percentage of yeast cells (approximately 10% for Pb60855 As*SCONC* A, approximately 5% for Pb60855 As*SCONC* B and approximately 30% for Pb01 As*SCONC* D). Nonetheless, the majority of cells were in intermediate phases and no mycelium cells were present in cultures with Pb60855 As*SCONC* A and Pb01 As*SCONC* D. Although our transcriptional data indicates that *SCONC* down-regulation would allow the yeast cells to consume inorganic sulfur compounds by the de-repression of the corresponding metabolic pathway, a complete transition was not observed in the absence of organic sulfur compounds.

**Figure 4:**
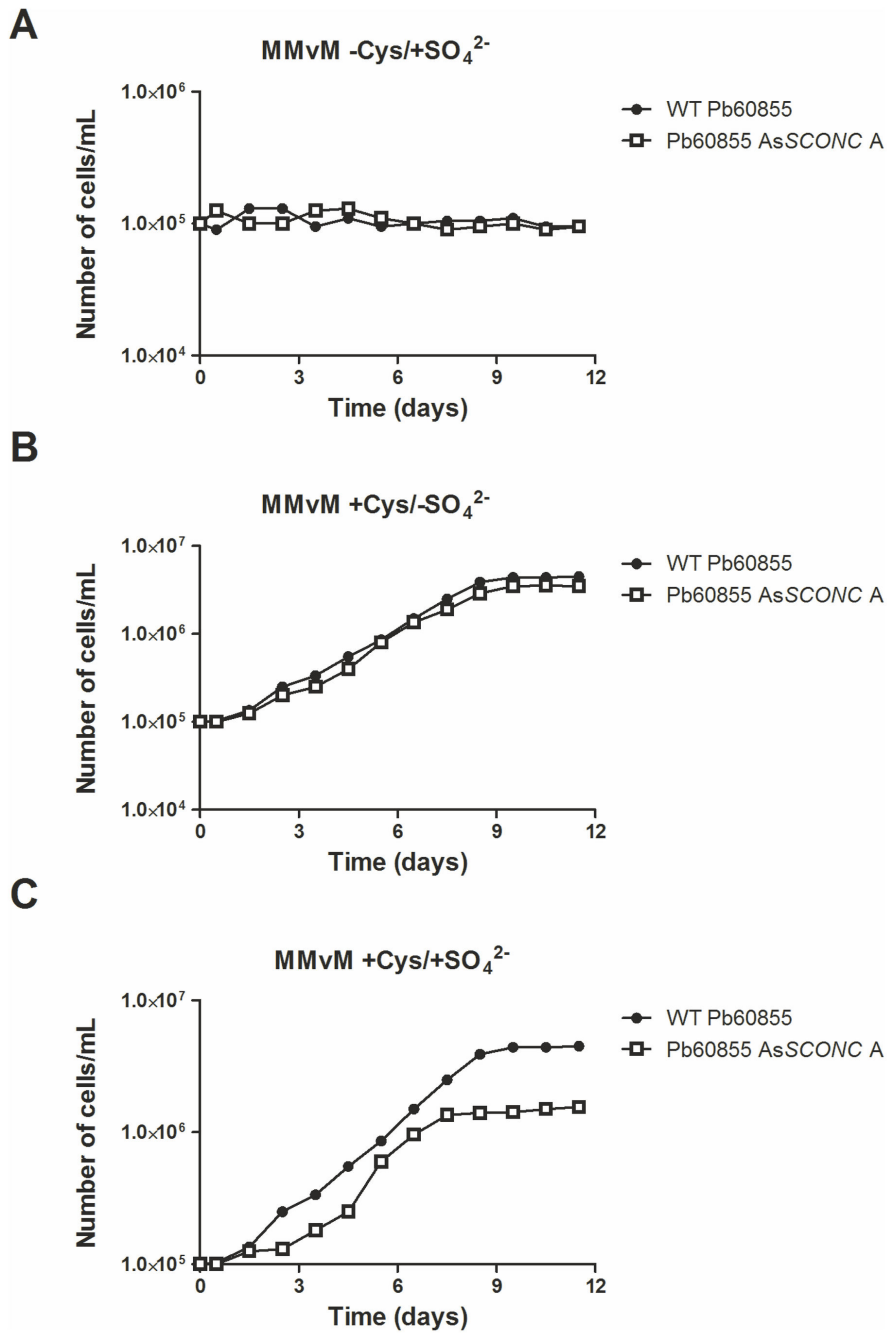
*P. brasiliensis* AsSCONC transformants do not grow in medium supplemented with inorganic sulfur sources only. *P. brasiliensis* AsSCONC transformants are unable to grow in medium supplemented with inorganic sulfur sources only (MMvM -Cys/+SO_4_^2-^) and do not sustain yeast growth together with low biomass yield in medium with both organic and inorganic sulfur sources (MMvM +Cys/+SO_4_^2-^). Pb60855 and Pb60855 As*SCONC* A yeast cells were grown in complete MMvM (MMvM +Cys/+SO_4_^2-^), MMvM without inorganic sulfur supplementation (MMvM +Cys/-SO_4_^2-^) and MMvM without organic sulfur compounds supplementation (MMvM -Cys/+SO_4_^2-^) at 37°C and samples were collected at specific time points to determine growth curves.

To investigate if *SCONC* silencing was affecting the growth of *Paracoccidioides* yeast cells, we next analyzed the growth profiles of Pb60855 and Pb60855 As*SCONC* A in the three media previously mentioned. We have chosen to perform these assays with Pb60855 As*SCONC* A as it was the transformant with the lowest *SCONC* expression. As shown in Figure 4.A, P *. brasiliensis* was not able to surpass its auxotrophy to organic sulfur sources, even when *SCONC* was down-regulated. Concomitantly, when in the presence of organic sulfur compounds, both wild-type Pb60855 and As*SCONC* A showed similar growth patterns, indicating that inorganic sulfur compounds are not required for the growth of the yeast phase (Figure 4.B). However, when both organic and inorganic sulfur sources were present in the medium, Pb60855 As*SCONC* A revealed a significantly lower growth rate and final optical density compared to the wild-type strain (Figure 4.C), indicating that inorganic sulfur was having a negative effect on the biomass yield.

### Silencing of *SCONC* reduces cellular ATP levels and NADPH pool thus increasing ROS accumulation

**Figure 5:**
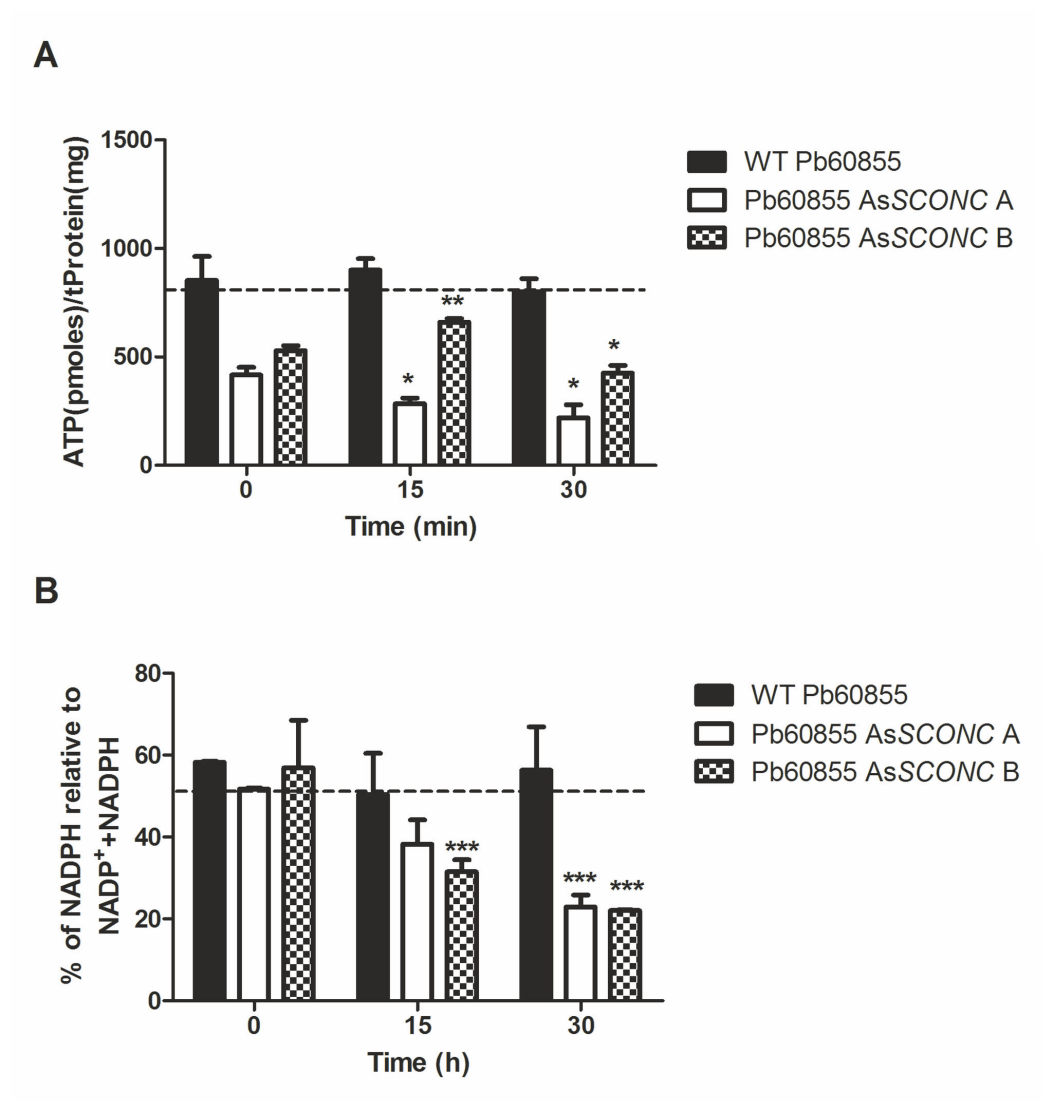
Down-regulation of *SCONC* impairs the ATP/NADPH pool in the presence of inorganic sulfur compounds. (**A**) ATP content in wild-type strain Pb60855, Pb60855 As*SCONC* A and Pb60855 As*SCONC* B, cultured at 37°C to the exponential growth phase in MMvM without inorganic sulfur compounds supplementation (MMvM +Cys/-SO_4_^2-^) at time 0, and 15 and 30 min after a pulse with inorganic sulfur [MgSO_4_.7H_2_O (2 mM) and (NH_4_)_2_SO_4_ (15 mM)]. Asterisks represent significant differences between 0 min and either 15 min or 30 min for each strain (*p<0.05; ***p<0.01); (**B**) % of NADPH relative to NADP^+^+NADPH in wild-type strain Pb60855, Pb60855 As*SCONC* A and Pb60855 As*SCONC* B cultured at 37°C to the exponential growth phase in MMvM without inorganic sulfur compounds supplementation (MMvM +Cys/-SO_4_^2-^) at time 0, and 15 and 30 min after a pulse with inorganic sulfur [MgSO_4_.7H_2_O (2 mM) and (NH_4_)_2_SO_4_ (15 mM)]. Asterisks represent significant differences between 0 min and either 15 min or 30 min for each strain (***p<0.001). Bar graphs indicate mean and standard deviation in three independent experiments. Statistical analysis was performed comparing 15 and 30 min to time 0 for each clone.

As the growth rate and final optical density in complete MMvM were lower in cultures of the As*SCONC* transformants compared to wild-type cultures (Figure 4.C), we hypothesized that inorganic sulfur metabolism was leading to a reduction on the ATP and NADPH cellular pools. To confirm this hypothesis, both wild-type Pb60855 and Pb60855 As*SCONC* A and B strains were cultured in MMvM without inorganic sulfur compounds and pulsed with inorganic sulfur [MgSO_4_.7H_2_O (2 mM) and (NH_4_)_2_SO_4_ (15 mM)]. Intracellular levels of ATP and NADPH were subsequently measured after 0, 15 and 30 min. The ATP and NADPH pool was lower in the As*SCONC* transformants at basal levels (0 min) than the observed for the wild-type strain (Figures 5.A and 5.B), except for the NADPH levels in Pb60855 As*SCONC* B transformant. The pulse with inorganic sulfur compounds resulted in a significant decrease of the ATP and NADPH levels in the As*SCONC* transformants, while those of the wild-type strain remained constant (Figures 5.A and 5.B). Taking into consideration the ATP and NADPH requirements for the inorganic sulfur metabolism, these results are consistent with the metabolic ability of the As*SCONC* transformants to use inorganic sulfur compounds.

**Figure 6:**
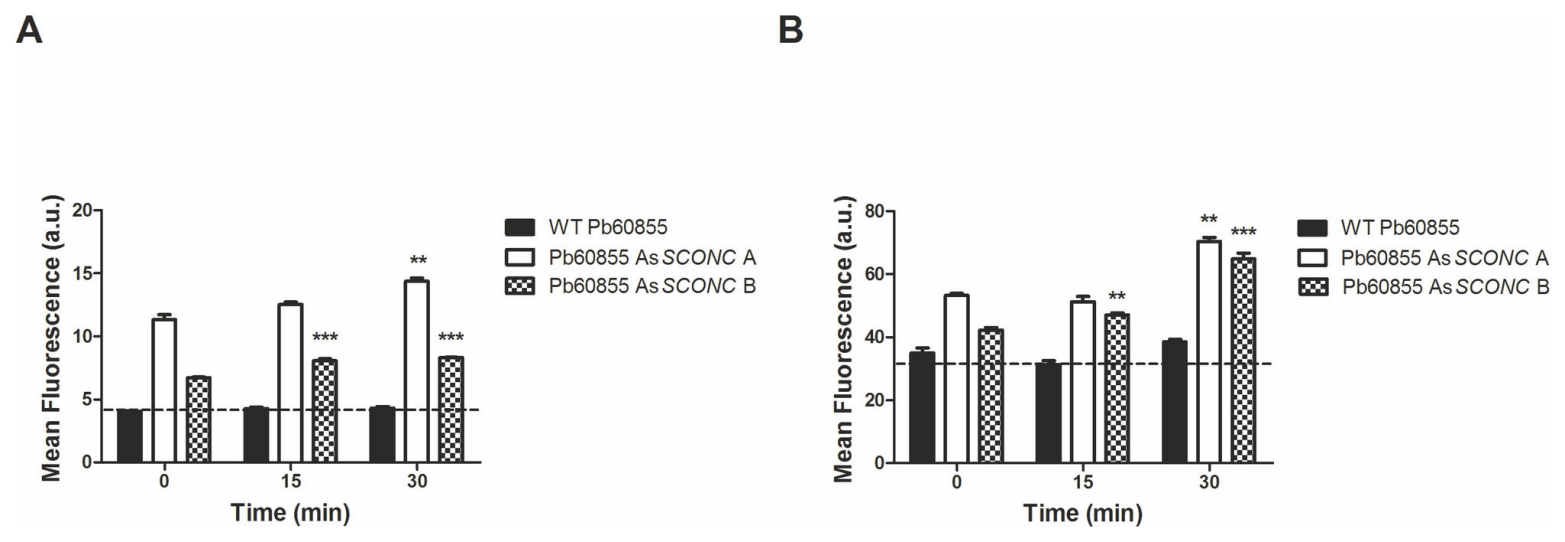
As*SCONC* transformants present high intracellular levels of reactive oxygen species. (**A**) FACS measurements of superoxide anions using the probe dihydroethidium (DHE) in wild-type strain Pb60855, Pb60855 As*SCONC* A and Pb60855 As*SCONC* B, cultured at 37°C in MMvM without inorganic sulfur compounds supplementation (MMvM +Cys/-SO_4_^2-^) at time 0 and 15 and 30 min after a punch with inorganic sulfur compounds. Asterisks represent significant differences between 0 min and either 15 min or 30 min for each strain (**p<0.01; ***p<0.001); (**B**) FACS measurements of H_2_O_2_ using dihydrorhodamine 123 (DHR) in wild-type strain Pb60855, Pb60855 As*SCONC* A and Pb60855 As*SCONC* B, cultured at 37°C in MMvM without inorganic sulfur compounds supplementation (MMvM +Cys/-SO_4_^2-^) at time 0 and 15 and 30 min after a pulse with inorganic sulfur compounds [MgSO_4_.7H_2_O (2 mM) and (NH_4_)_2_SO_4_ (15 mM)]. Asterisks represent significant differences between 0 min and either 15 min or 30 min for each strain (**p<0.01; ***p<0.001). Bar graphs indicate mean and standard deviation of fluorescence/cell (arbitrary units) measured in 1x10^5^ cells per sample in three independent experiments. Statistical analysis was performed comparing 15 and 30 min to time 0 for each clone.

The cellular NADPH pool is critical for the maintenance of the reduced glutathione pool and thus for the maintenance of the cellular redox balance. Therefore, we next evaluated the accumulation of ROS such as superoxide anions and H_2_O_2_ in the wild-type strain and As*SCONC* transformants after a pulse with inorganic sulfur compounds [MgSO_4_.7H_2_O (2 mM) and (NH_4_)_2_SO_4_ (15 mM)]. This was performed by FACS analysis using dihydroethidium (DHE) and dihydrorhodamine 123 (DHR), respectively, as fluorescence markers [31]. We found that both As*SCONC* transformants presented higher levels of superoxide anions and H_2_O_2_ at time 0 than those of the wild-type strain (Figures 6.A and 6.B). Moreover, there was a statistically significant increase of both ROS species overtime in the As*SCONC* transformants upon the inorganic sulfur pulse, while in the wild-type strain ROS levels were maintained (Figures 6.A and 6.B). These results reveal that by diverting NADPH to sulfur metabolism, As*SCONC* cells accumulate more ROS such as H_2_O_2_ (Figure 6.B). The alteration of the cellular redox balance in these cells most probably leads to an amplification loop of ROS generation that, in addition to the uncoupling of oxidative phosphorylation during *P. brasiliensis* mycelium-to-yeast transition, accounts for the observed increased levels of superoxide anions (Figure 6.A).

### SconCp contributes to the *in vivo* virulence of *P. brasiliensis*

**Figure 7:**
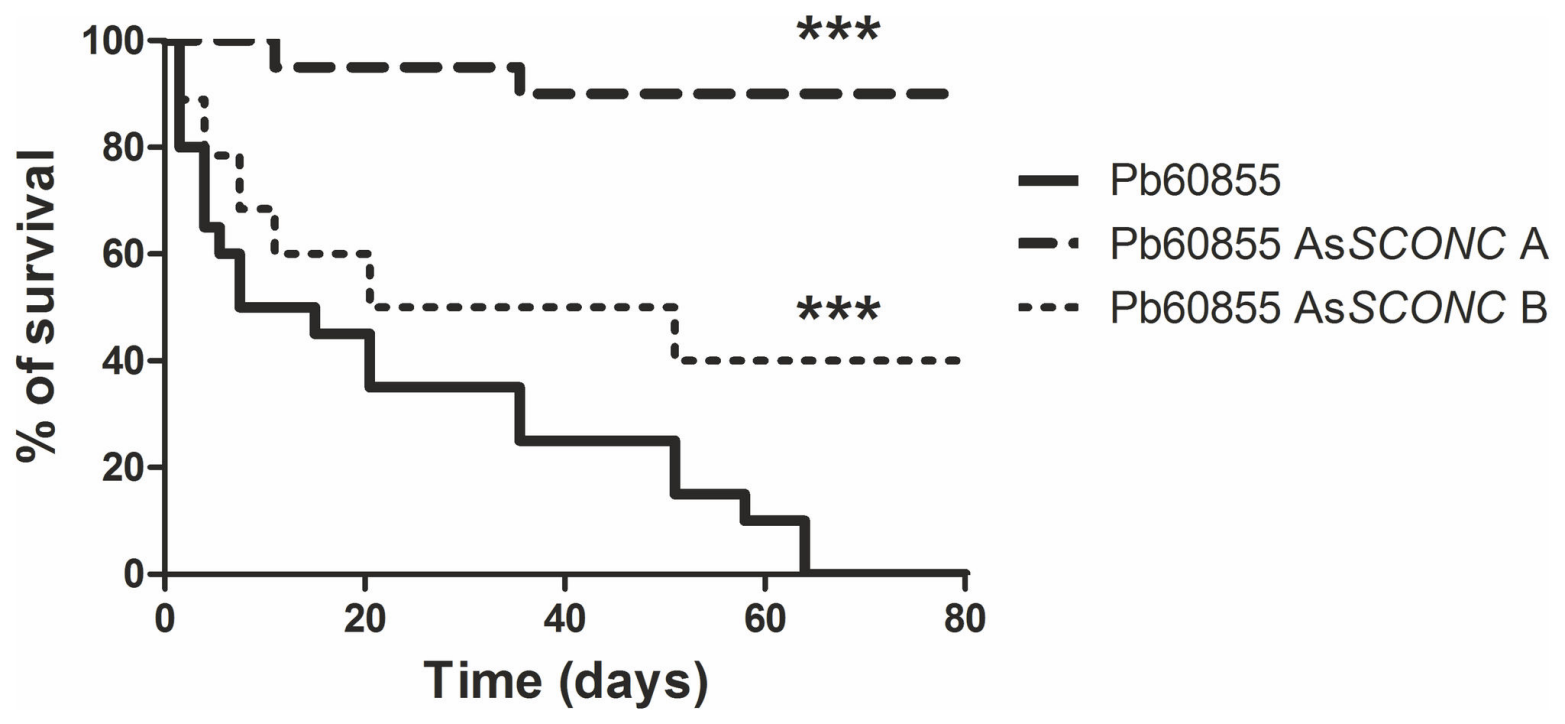
Silencing of *SCONC* decreases virulence of *P. brasiliensis* yeast cells. Representative survival curves of an experimental i.v. infection carried out in C57BL/6 mice (n=20) with 1x10^6^ wild-type Pb60855, Pb60855 As*SCONC* A and Pb60855 As*SCONC* B yeast cells grown to the exponential phase in BHI. Asterisks represent significant differences between mice infected with Pb60855 and mice infected with either Pb60855 As*SCONC* A or Pb60855 As*SCONC* B.

So far our data clearly implicate SconCp in *Paracoccidioides* mycelium-to-yeast morphological switch, thus making SconCp a good candidate for a virulence factor of this fungus. To address this question, C57BL/6 male mice were intravenously infected with 1X10^6^ exponential yeast cells of wild-type Pb60855 strain and its respective As*SCONC* transformants. We found that 67 days post-infection all mice infected with the wild-type strain had succumbed (Figure 7), which is in accordance with the virulence pattern normally observed for this strain. In contrast, Pb60855 As*SCONC* B transformant revealed an intermediate virulence level in what regards the survival of the infected mice (Figure 7). Mice infected with Pb60855 As*SCONC* A transformant survived up to 80 days post-infection (Figure 7), suggesting a less virulent phenotype for this transformant. As observed before, the phenotype of each transformant when compared to the wild-type strain is correlated with the level achieved for *SCONC* down-regulation (which was highest for transformant A). Our results strongly suggest SconCp as an essential player on *P. brasiliensis* species virulence. 

## Discussion

Recent studies uncovered several genes involved in the pathogenicity of *P. brasiliensis* and its degree of virulence, including *CDC42* [26,32], *GP43* [33-35], *HAD32* [36] and *AOX* [37]. However, the research on *P. brasiliensis* genetic determinants that govern conidia/mycelium-to-yeast transition and subsequently fungal virulence has been highly neglected. The understanding of the mechanisms underlying the morphological transition of *P. brasiliensis* from the conidia/mycelial phase to the pathogenic yeast phase is essential, as this is critical for the establishment and development of paracoccidioidomycosis. In the last decades, the only known factor attributed to this morphological switch was the temperature shift that occurs once *P. brasiliensis* conidia/mycelium reach the lungs of the host [1,38,39].

In this study we evaluated the effect of SconCp, the negative regulator of the inorganic sulfur assimilatory pathway, in *P. brasiliensis* dimorphism and virulence. Knowing from previous studies that *SCONC* is highly expressed in the yeast phase *of P. brasiliensis*, and that it blocks the assimilation of inorganic sulfur sources [13-16], we down-regulated *Paracoccidioides* *SCONC* using a gene silencing approach [26]. The reduction of *SCONC* expression levels led to the up-regulation of several genes belonging to the inorganic sulfur pathway. These results are in line with data obtained for other fungi. Mutations in *sconC1* and *sconC2* genes from *Aspergillus nidulans* were shown to impair methionine-mediated sulfur metabolite repression, allowing the fungus to the produce sulfur-metabolism related enzymes [30,40]. Characterization of a *Neurospora crassa* mutant in *SCON-2* gene also revealed a de-repression of the sulfur metabolism, contrarily to the wild-type strain [29]. In *Saccharomyces cerevisiae*, mutations in *MET30* (the homolog of *SCONC* from *P. brasiliensis*) revealed to impair the repression of the sulfur network [41].

Knowing that the conidia/mycelium-to-yeast transition is a requisite for the development of paracoccidioidomycosis [1], we tested whether down-regulation of *SCONC* and consequent up-regulation of inorganic sulfur pathway-related genes impacted on the dimorphic process. We found that upon de-repression of the inorganic sulfur pathway by down-regulation of *SCONC*, the yeasts’ auxotrophy for organic sulfur sources was surpassed. Consequently, the mycelium-to-yeast transition in media supplemented only with inorganic sulfur sources was possible. These data further support a regulatory role for SconCp during the dimorphic process, by repressing inorganic sulfur metabolism related genes.

Concomitantly, a similar occurrence was found upon down-regulation of *SCONC* in *Paracoccidioides* species other than *P. brasiliensis*, such as in *P* *. lutzii* species. This is a good indicator that, although great sequence and morphological divergences separate these species, *P. brasiliensis* and *P* *. lutzii* share the same regulatory sulfur mechanisms and dimorphic traits.

Although *P. brasiliensis* cells were able to assimilate inorganic sulfur sources, they could not accomplish a complete transition in the absence of organic sulfur compounds. Together with this, the fact that down-regulated transformants presented lower biomass yields in medium supplemented with both inorganic and organic sulfur sources, led us to question if the assimilation of inorganic sulfur was causing a metabolic imbalance, thereby affecting growth of *P. brasiliensis* yeast cells. The high demand of ATP and NADPH for inorganic sulfur metabolism (Figure 2.A) could be hampering both the energy available for cellular processes and the ability of *P. brasiliensis* to scavenge ROS. Our results show that the usage of inorganic sulfur sources in As*SCONC* transformants indeed decreased the availability of total NADPH overtime. This fact, together with the natural low activity of glucose-6-phosphate dehydrogenase in the yeast phase of *P. brasiliensis*, the main cellular source of NADPH [42], could explain the high accumulation of H_2_O_2_ and superoxide anions in As*SCONC* transformants. This is possibly resulting in a decrease of the reduced glutathione pool, thus impairing the cellular redox balance. In fact, an interplay between the sulfur assimilatory pathway and the formation of the reduced form of glutathione, a powerful anti-oxidant [43], is well described in some fungi [44,45]. A similar observation was reported upon selenium uptake by *S. cerevisiae* cells. Selenium metabolites share a highly similar chemical and physical nature with sulfur metabolites, and are both thought to follow the same metabolic routes. In conditions of sulfur deficiency, selenium uptake by *S. cerevisiae* cells led to an intracellular redox imbalance, linked to a disproportionate ration between the reduced form of glutathione and the oxidized one, a circumstance shown to be detrimental for cell viability [46].

As for an implication of the inorganic sulfur metabolism in the energy available for cellular processes, we were able to detect a decrease on the intracellular pool of ATP in the As*SCONC* transformants. Since the uncoupling of oxidative phosphorylation during *P. brasiliensis* mycelium-to-yeast transition is known to reduce the levels of ATP [10], the remaining ATP pool in the As*SCONC* transformants is likely not fulfilling the cellular requirements upon inorganic sulfur metabolism. Taken together, our findings on the alterations of the cellular pools of both NADPH and ATP can be accountable for the low biomass yields of the As*SCONC* transformants, and their inability to grow on the yeast phase using only inorganic sulfur sources.

Finally we also show that the down-regulation of *SCONC* profoundly alters the outcome of the infection in an *in vivo* mouse model of infection. In fact, the As*SCONC* transformants were less virulent to mice, being the degree of virulence correlated with the efficiency of *SCONC* silencing.

Therefore, our data suggest a novel role for SconCp as a virulence factor for *P. brasiliensis*. Lack of virulence of *SCONC* down-regulated transformants is likely due to an impairment of the cells’ antioxidant properties and energy in the form of ATP, as discussed herein. Since the silencing of this molecule can in fact abrogate the *in vivo* virulence of *P. brasiliensis*, it will be essential to explore the modulation of SconCp in *P. brasiliensis* as a tool to obtain an attenuated vaccine, and also possible ways to abrogate the expression or the activity of SconCp in a therapeutic perspective.

## Supporting Information

Figure S1 Down-regulation of *SCONC* in *P. brasiliensis* allows mycelium-to-yeast transition in the absence of organic sulfur compounds. Evaluation of Pb60855 As*SCONC* B morphotypes during: (**A**) Yeast-to-mycelium transition at 26°C in complete MMvM (MMvM +Cys/+SO_4_^2-^), MMvM without inorganic sulfur compounds supplementation (MMvM +Cys/-SO_4_^2-^) and MMvM without organic sulfur compounds supplementation (MMvM -Cys/+SO_4_^2-^); (**B**) Mycelium-to-yeast transition at 37°C in complete MMvM (MMvM +Cys/+SO_4_^2-^), MMvM without inorganic sulfur compounds supplementation (MMvM +Cys/-SO_4_^2-^) and MMvM without organic sulfur compounds supplementation (MMvM -Cys/+SO_4_^2-^).
(TIF)

Figure S2 Down-regulation of *SCONC* in *P* *. lutzii* allows mycelium-to-yeast transition in the absence of organic sulfur compounds. Evaluation of Pb01 and Pb01 As*SCONC* D morphotypes during: (**A**) Yeast-to-mycelium transition at 26°C in complete MMvM (MMvM +Cys/+SO_4_^2-^), MMvM without inorganic sulfur compounds supplementation (MMvM +Cys/-SO_4_^2-^) and MMvM without organic sulfur compounds supplementation (MMvM -Cys/+SO_4_^2-^); (**B**) Mycelium-to-yeast transition at 37°C in complete MMvM (MMvM +Cys/+SO_4_^2-^), MMvM without inorganic sulfur compounds supplementation (MMvM +Cys/-SO_4_^2-^) and MMvM without organic sulfur compounds supplementation (MMvM -Cys/+SO_4_^2-^).
(TIF)
