# Supplementary figures and images for: P. brasiliensis Virulence Is Affected by SconC, the Negative Regulator of Inorganic Sulfur Assimilation
Source: PLoS One. 2013 Sep 16;8(9):e74725. doi: 10.1371/journal.pone.0074725 (PMC3774720; doi:10.1371/journal.pone.0074725)

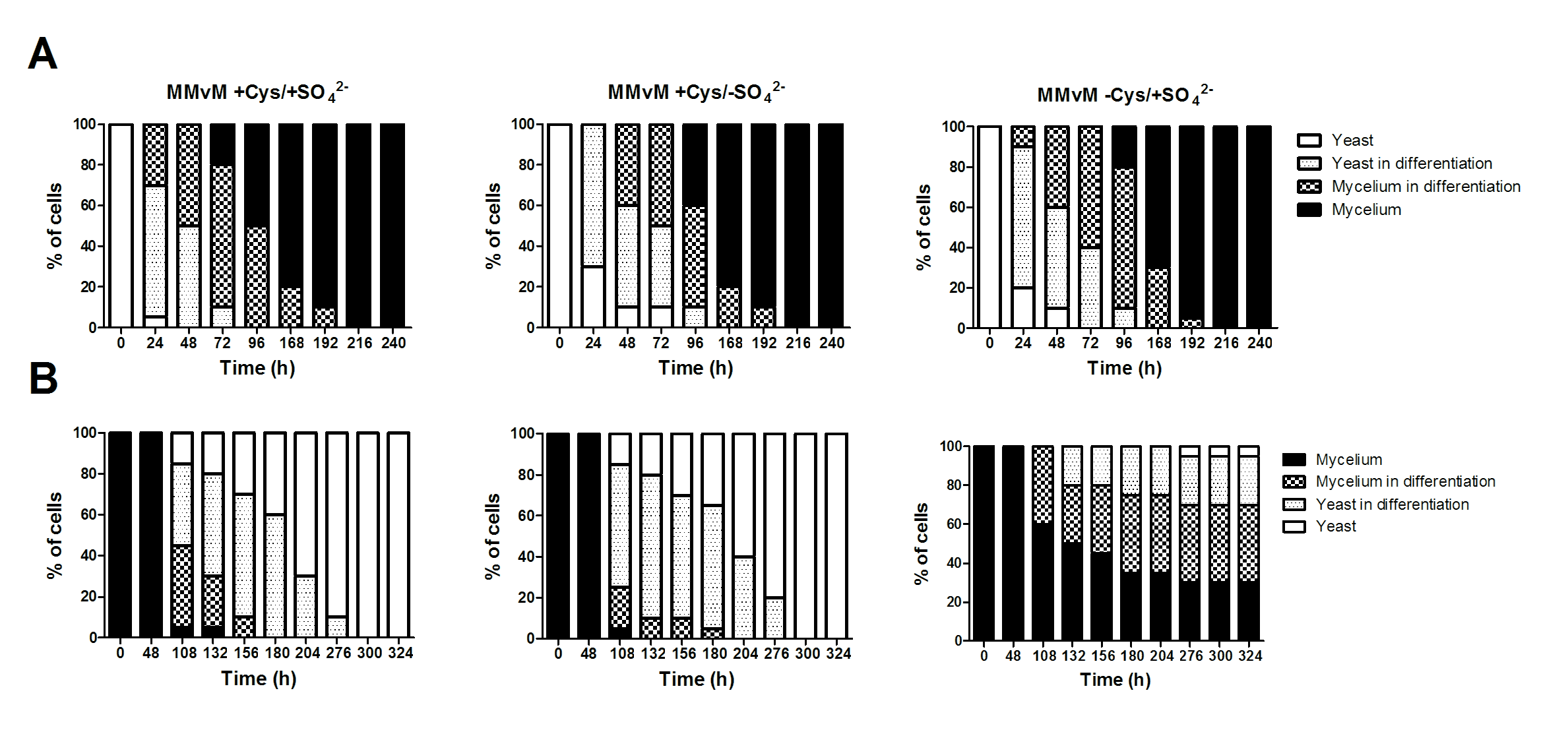

Supplement: Figure S1 — Down-regulation of SCONC in P. brasiliensis allows mycelium-to-yeast transition in the absence of organic sulfur compounds. Evaluation of Pb60855 AsSCONC B morphotypes during: (A) Yeast-to-mycelium transition at 26°C in complete MMvM (MMvM +Cys/+SO4 2-), MMvM without inorganic sulfur compounds supplementation (MMvM +Cys/-SO4 2-) and MMvM without organic sulfur compounds supplementation (MMvM -Cys/+SO4 2-); (B) Mycelium-to-yeast transition at 37°C in complete MMvM (MMvM +Cys/+SO4 2-), MMvM without inorganic sulfur compounds supplementation (MMvM +Cys/-SO4 2-) and MMvM without organic sulfur compounds supplementation (MMvM -Cys/+SO4 2-). (TIF) [file pone.0074725.s001.tif]

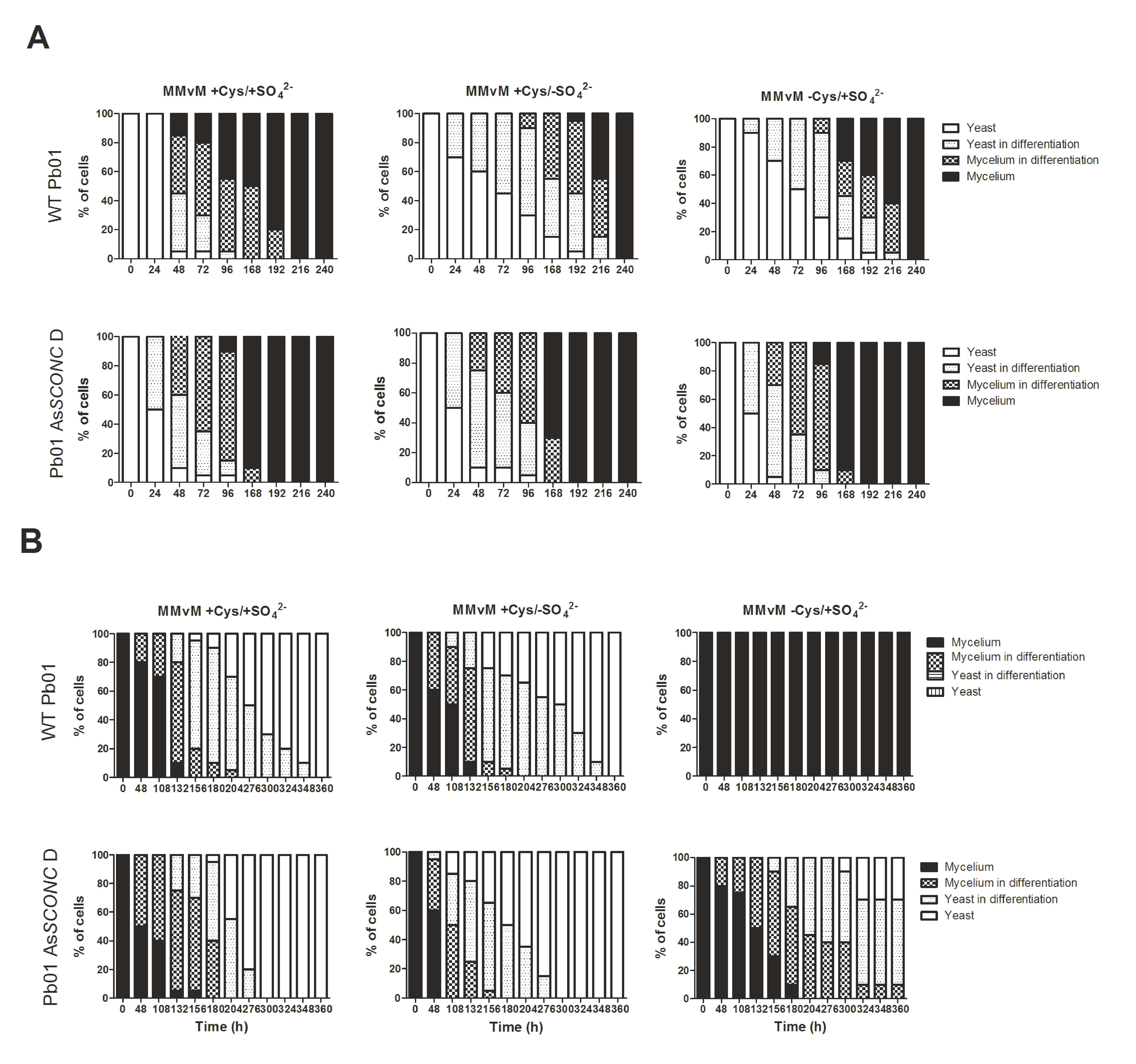

Supplement: Figure S2 — Down-regulation of SCONC in P . lutzii allows mycelium-to-yeast transition in the absence of organic sulfur compounds. Evaluation of Pb01 and Pb01 AsSCONC D morphotypes during: (A) Yeast-to-mycelium transition at 26°C in complete MMvM (MMvM +Cys/+SO4 2-), MMvM without inorganic sulfur compounds supplementation (MMvM +Cys/-SO4 2-) and MMvM without organic sulfur compounds supplementation (MMvM -Cys/+SO4 2-); (B) Mycelium-to-yeast transition at 37°C in complete MMvM (MMvM +Cys/+SO4 2-), MMvM without inorganic sulfur compounds supplementation (MMvM +Cys/-SO4 2-) and MMvM without organic sulfur compounds supplementation (MMvM -Cys/+SO4 2-). (TIF) [file pone.0074725.s002.tif]
